# Altered expression of fragile X mental retardation-1 (FMR1) in the thymus in autoimmune myasthenia gravis

**DOI:** 10.1186/s12974-021-02311-y

**Published:** 2021-11-17

**Authors:** Scott Thomas, Odessa-Maud Fayet, Frédérique Truffault, Elie Fadel, Bastien Provost, Abderaouf Hamza, Sonia Berrih-Aknin, Jean-Paul Bonnefont, Rozen Le Panse

**Affiliations:** 1 Sorbonne University, INSERM, Institute of Myology, Center of Research in Myology, F-75013 Paris, France; 2 Marie Lannelongue Hospital, Paris-Sud University, Le Plessis-Robinson, France; 3 Université de Paris, Institut Imagine UMR1163, Hôpital Universitaire Necker-Enfants Malades, AP-HP Paris, France

**Keywords:** Fragile X syndromes, Autoimmunity, FMR1, Thymus, Myasthenia gravis, Inflammatory cytokines, Thymic epithelial cells, CTCF, Max

## Abstract

Predisposition to autoimmunity and inflammatory disorders is observed in patients with fragile X-associated syndromes. These patients have increased numbers of CGG triplets in the 5’ UTR region of *FMR1* (Fragile X Mental Retardation 1) gene, that affects its expression. *FMR1* is decreased in the thymus of myasthenia gravis (MG) patients, a prototypical autoimmune disease. We thus analyzed the number of CGG triplets in *FMR1* in MG, and explored the regulatory mechanisms affecting thymic *FMR1* expression. We measured the number of CGGs using thymic DNA from MG and controls, but no abnormalities in CGGs were found in MG that could explain thymic decrease of *FMR1*. We next analyzed by RT-PCR the expression of *FMR1* and its transcription factors in thymic samples, and in thymic epithelial cell cultures in response to inflammatory stimuli. In control thymuses, *FMR1* expression was higher in males than females, and correlated with *CTCF* (CCCTC-binding factor) expression. In MG thymuses, decreased expression of *FMR1* was correlated with both *CTCF* and *MAX* (Myc-associated factor X) expression. Changes in *FMR1* expression were supported by western blot analyses for FMRP. In addition, we demonstrated that *FMR1, CTCF* and *MAX* expression in thymic epithelial cells was also sensitive to inflammatory signals. Our results suggest that *FMR1* could play a central role in the thymus and autoimmunity. First, in relation with the higher susceptibility of females to autoimmune diseases. Second, due to the modulation of its expression by inflammatory signals that are known to be altered in MG thymuses.

## Introduction

Regulation of the fragile X mental retardation-1 (*FMR1*) gene is dependent on CGG triplets in its 5’ UTR region. In most individuals the number of CGG triplets is around 30 ranging from 26 to 32 depending on studies [1]. However, this region can become unstable leading to CGG triplet expansion associated with diseases such as the fragile X syndrome (FXS, above 200 CGGs), fragile X-associated tremor/ataxia syndrome (FXTAS) or fragile X premature ovarian insufficiency syndrome (FXPOI) (from 55 to 200 CGGs) [1]. Individuals with 41 to 54 CGGs are considered in the “gray zone” genotype and seem also more at risk to have FXPOI [2]. Recent studies also define a new category of individuals with a lower number of CGGs, usually below 24–25. This genotype is also associated with health problems [3, 4] and a lower *FMR1* expression [5]. *FMR1* codes for the fragile X mental retardation protein (FMRP), a RNA binding protein with many functions. However, its role has been especially investigated in the nervous system [6], despite the fact that FMRP is highly expressed in other adult tissues, such as in the thymus [7].

Immune-mediated disorders, in particular autoimmune diseases, have been reported in premutation carriers, such as autoimmune thyroid disorders, rheumatoid arthritis, systemic lupus erythematosus, Sjögren’s syndrome [8, 9]. Myasthenia gravis (MG) is a rare autoimmune disease characterized by invalidating muscle weaknesses. It is caused by autoantibodies targeting components of the neuromuscular junction, mainly the acetylcholine receptor (AChR) [10]. We previously demonstrated that *FMR1* expression is decreased in MG thymus [11]. The thymus plays a key role in central tolerance mechanisms avoiding the escape of autoreactive T cells and autoimmunity [12]. In addition, in MG, the thymus is often abnormal, and is characterized in AChR^+^ MG by ectopic germinal centers developing in an inflammatory environment [10]. These observations prompted us to further investigate the potential role of FMR1 in MG.

We hypothesized that MG patients could have an abnormal number of CGGs that would affect thymic *FMR1* expression. This genotype could predispose to MG and even autoimmunity. Here, we analyzed the number of CGG triplets in the thymus of MG patients as compared to controls, and investigated the mechanisms regulating FMR1 expression in the thymus.

## Methods

### Human samples

For DNA analyses, thymic biopsies from AChR^+^ MG patients without thymoma (*n* = 58 females and 22 males, 18 to 50 years old) were collected after thymectomy and control thymic biopsies (*n* = 38 females and 10 males, 2 days to 50 years old) were collected from donors undergoing cardiovascular surgery at the Marie Lannelongue Surgical Center (Le Plessis-Robinson, France). In MG patients, peripheral mononuclear blood cells (PBMCs) (n = 8 females and 1 male) were also isolated from fresh whole blood, collected in EDTA tubes, using the Ficoll technique.

For RT-PCR analyses, thymic biopsies were also collected from AChR^+^ MG patients (*n* = 12 females, 15–35 years old) and control thymic biopsies (infant females n = 6, 3–12 months; adult females *n* = 6, 15–33 years old, and adult males *n* = 6, 15–44 years old). MG patients included had either a low-grade thymic hyperplasia (with 2 or fewer GCs per section, *n* = 6) or a high-grade thymic hyperplasia (with 3 or more GCs per section, *n* = 6). For western blot analyses, thymic biopsies were from AChR^+^ MG patients (*n* = 3 females, 31–37 years old) and control thymic biopsies (females (*n* = 2) and males (*n* = 2), 25–49 years old). All MG patients were only treated with cholinesterase inhibitors and had no other known diseases including thymoma.

Studies on blood and thymic samples were approved by local ethics committees (RCB 2006-A00164-47 and RCB 2010-A00250-39).

### DNA extraction and CGGs analysis

DNA extraction was done from thymus biopsies or PBMCs using the all prep ADN/ARN mini kit from Qiagen (Courtaboeuf, France). Analysis of CGG repeat length and the number of AGG interruptions were determined using the AmplideX® *FMR1* PCR kit from Asuragen (Theradiag, Marne la Vallée, France) according to the manufacturer instructions. Results were analyzed on GeneMapper software.

### RT-PCR

RT-PCR analyses were done as previously described [11]. The primer sequences were from Eurogentec (Angers, France): *FMR1* (F: 5’-CAGGGCTGAAGAGAAGATGG-3’, R: 5’-ACAGGAGGTGGGAATCTGA-3’), *CTCF* (F: 5’-ACCAGTGGAGAATTGGTTCG, R: 5’-TCATGTGCCTTTTCAGCTTG-3’), *MAX* (F: 5’-ATGACATCGAGGTGGAGAGC-3’, R: 5’-CCTTGGAGTGATGGGACTGA-3’). PCR were normalized to *28S* (F: 5’-GGTAGGGACAGTGGGAATCT-3’, R: 5’-CGGGTAAACGGCGGGAGTAA-3’) or *GAPDH* (F: 5’-CGACCACTTTGTCAAGCTCA-3’, R: 5’-AGGGGTCTACATGGCAACTG-3’).

### Western blot

Total thymic proteins were extracted in a solution containing 5% Tris–HCl 20 mM (pH 7.4), 0.1% Triton X100 supplemented with Halt™ Protease Inhibitor Cocktail (ThermoFisher Scientific, Villebon-sur-Yvette, France) using the fast prep apparatus. Extracts were cleared by centrifugation (13,000 g, 10 min. 4 °C). 20 µg of proteins were separated by 10% SDS-polyacrylamide gels and transferred onto nitrocellulose membranes. Membranes were blocked for 2 h in 5% dried milk in TBST (0.1% Tween-20 in Tris-buffered saline) and incubated overnight at 4 °C in TBST-3% dried milk with an anti-FMRP antibody (1:1000; Clone 6B8/FMRP, Biolegend, Amsterdam, The Netherlands) or an anti-GAPDH antibody for 1 h (1:10,000, Clone 6C5, CliniSciences, Nanterre, France). Membranes were washed in TBST and incubated for 1 h with a StarBright Blue 700 goat anti-mouse IgG (1:10,000, Bio-Rad, Marnes-la-Coquette, France) in TBST-3% dried milk. The membranes were washed in TBST before detection of the immune signal using ChemiDoc™ imaging system (Bio-Rad).

### Thymic epithelial cell (TEC) culture

Primary human TECs were cultured from infant thymus as previously described [11]. TECs were seeded (1.4 × 10^5^ cells/cm^2^) in RPMI-5% horse serum for 24 h and treated with Poly(I:C) (100 μg/ml; InvivoGen, Toulouse, France), IFN-I 1000 UI/ml (R&D Systems, Lille, France), IFN-II (IFN-γ (1000UI/ml; R&D systems) or IL-6 (10 ng/ml; R&D systems) in RPMI-0.5% horse serum for 24 h.

### Statistical analyses

For 2-by-2 comparisons, parametric (t-test), non-parametric (Mann–Whitney test) or paired (Wilcoxon test) tests were performed as specified in figure legends. Correlation analyses were performed using Spearman's correlation coefficient for non-Gaussian distributed variables, with a *p* < 0.05 considered significant. For certain analyses, mean ± SEM were given in the text.

## Results

### Comprehensive analyses of CGG triplets in MG patients

**Fig. 1:**
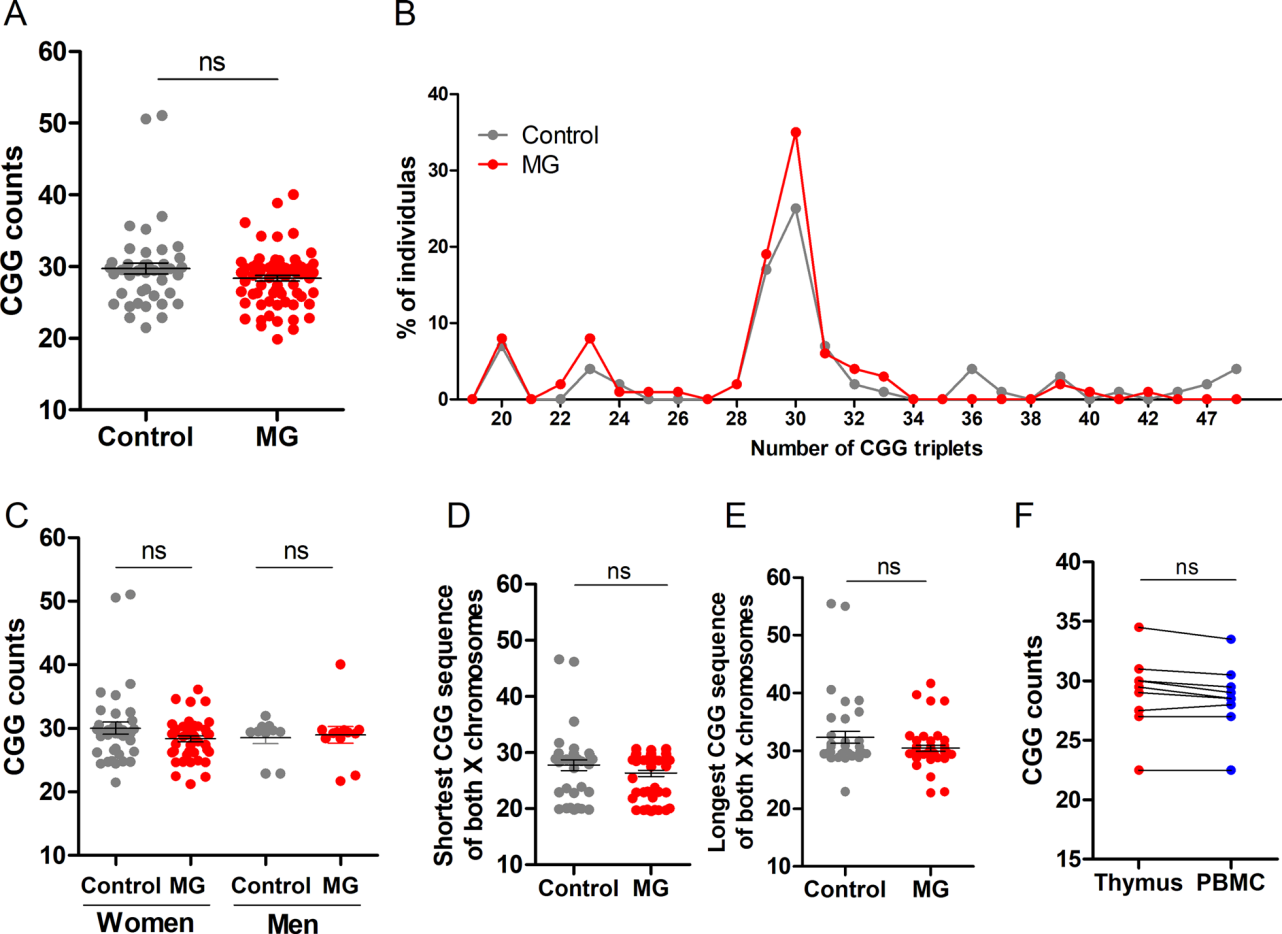
Analyses of CGG triplets in *FMR1* 5’UTR in the thymus of MG patients. Analysis of CGG repeat length using the AmplideX® FMR1 PCR kit. DNA was extracted from thymic biopsies. **A** Comparison of MG patients (*n* = 80) and non-MG patients (*n* = 48). Here to compare the number of CGG triplets in males and females, the mean of CGGs on both alleles of the X chromosome was calculated for females. **B** Distribution of the number of CGG triplets in MG patients and non-MG patients. **C** Comparison of female and male MG patients (n = 58 females and 22 males) and non-MG patients (*n* = 38 females and 10 males). **D**, **E** Separate analyses of the shorter **D** or longer **E** CGG sequences on each chromosome X in females. **F** Comparison of the number of CGG triplets on DNA extracted from thymic and blood cells from a same MG patients. *p* values were assessed with an unpaired *t*-test (**A**), a Mann–Whitney test (**C**–**E**), and a Wilcoxon test (**F**)

We analyzed the number of CGGs in 80 MG patients and 43 control donors. To compare the number of CGG triplets in males and females, the mean of CGGs on both alleles of the X chromosome was calculated for females. The control donor group had 2 premutated female carriers with 46/55 or 47/55 CGGs. The mean number of CGGs was 29.7 ± 0.8 (or 28.8 ± 0.5 without the premutated carriers). In MG patients, the mean number of CGG triplets was 28.4 ± 0.40. No significant difference was observed between controls and MG patients, and the distribution of the number of CGG triplets was similar with a peak of CGG triplets at 29–30 (Fig. 1A, B).

As females are more susceptible to autoimmunity, we analyzed separately female and male samples. We did not observe differences between MG and non-MG-donors in both groups (Fig. 1C). The number of CGG triplets can be different on each chromosome X inherited from the mother and the father. We analyzed separately the shorter (Fig. 1D) and the longer (Fig. 1E) CGG sequence of both chromosomes. However, we did not observe significant differences with this method of analysis. Variations of the number of CGGs for a given individual have been mentioned in different tissues [13]. To determine if variations could occur in the thymus, we compared the number of CGG triplets in DNA extracted from the thymus or PBMCs from the same donors. We showed that the number of CGGs was identical using DNA from the thymus or from PBMCs from MG patients (Fig. 1F).

The CGG repeat segment can be interrupted by AGG triplets, usually two AGGs in the general population [14]. In our samples, a few ones were difficult to interpret and were excluded. Analyzing 77 MG patients and 33 controls, the same proportion of AGG triplets were observed in both groups with 80% of the donors having two AGGs, 13% having one AGG and 7% three AGGs (data not shown). For patients with two AGG triplets, no difference in the number of CGG triplets was observed between controls and MG donors (data not shown).

### Differential expression of *FMR1* in the thymus in normal conditions and in MG patients

**Fig. 2:**
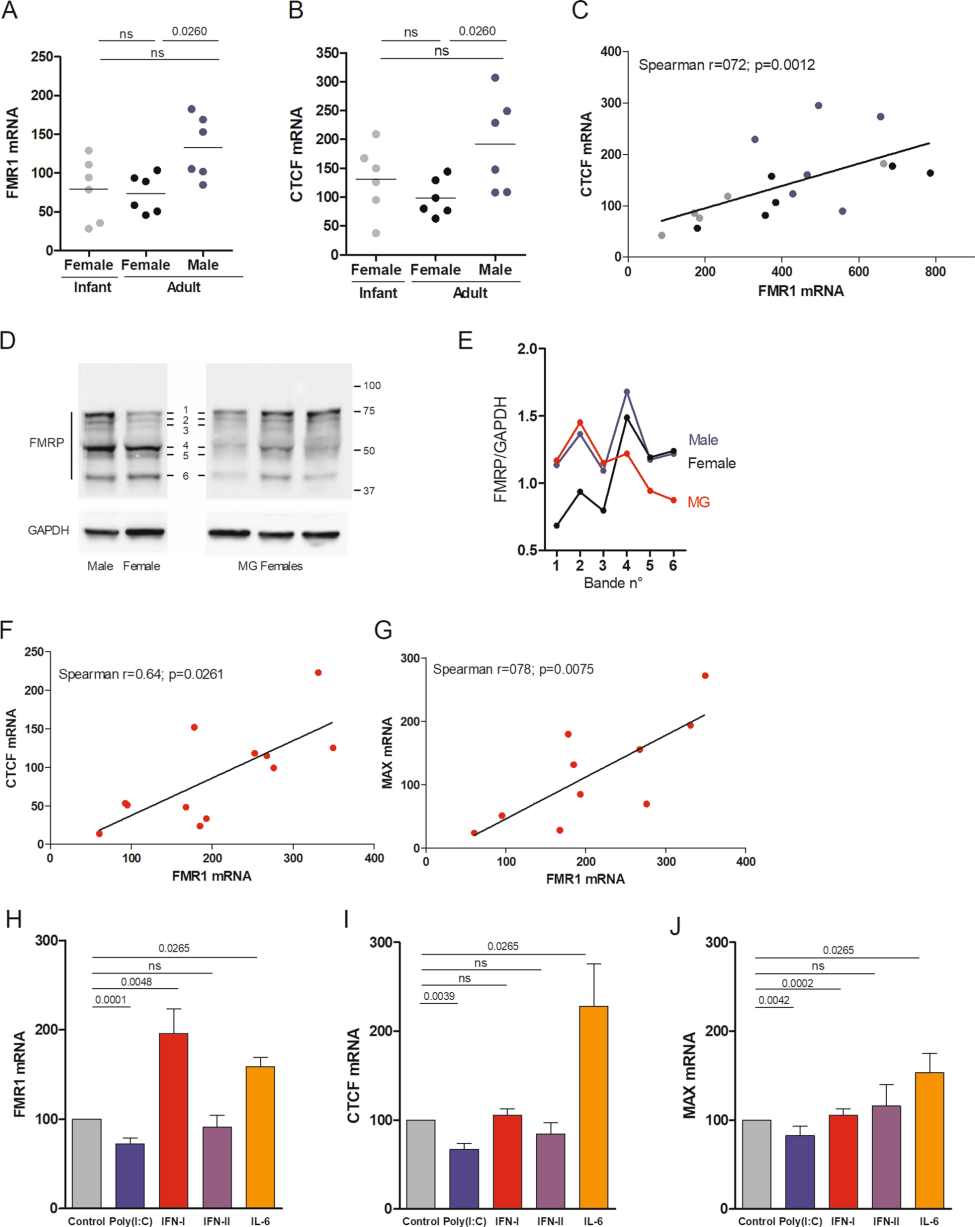
Analyses of *FMR1*, *CTCF* and *MAX* expression in the thymus and thymic epithelial cells. RT-PCR analysis for *FMR1* (**A**) and *CTCF* (**B**) in the thymus of non-MG donors (infant females (*n* = 6, grey dots), adult females (n = 6, black dots) and adult males (*n* = 6, bleu dots). Correlation between *FMR1* and *CTCF* mRNA expression in non-MG thymuses (**C**). Western blots for FMPR and GAPDH on thymic extracts. Six bands were recognized by the anti-FMRP antibody (**D**). Each band was quantified using Fiji and divided by the one corresponding to GAPDH. **E** for non-MG donors (female (*n* = 2) and male (*n* = 2) adults) and MG female patients (*n* = 3). RT-PCR analysis for *FMR1, CTCF and MAX* in the thymus non-MG female donors (infants and adults, *n* = 12, grey dots) and MG patients (*n* = 10–12, red dots). Correlation between *FMR1* and *CTCF* (**F**) or *MAX* (**G**) mRNA expression in MG thymuses. RT-PCR analysis for *FMR1* (**H**), *CTCF* (**I**) and *MAX* (**J**) in TECs from non-MG thymuses (*n* = 4–9 from different donors). TEC cultures were stimulated for 24 h with Poly(I:C) (100 μg/ml), IFN-I (1000 UI/ml), IFN-II (1000UI/ml) or IL-6 (10 ng/ml) in RPMI-0.5% horse serum for 24 h. PCRs with absolute quantification were performed for each gene analyzed and data were normalized to the GAPDH. For each experiment with a different TEC culture, the control values were set at 100. *p* values were assessed with a Mann–Whitney test and for correlation analyses a Spearman's correlation test was done

First, we analyzed the expression of *FMR1* mRNA in thymic samples from males and females. Primers used amplified all *FMR1* transcript variants and we clearly observed a significant higher expression in males compared to females (Fig. 2A). Human FMR1 gene has 17 exons that can undergo alternative splicing, resulting in different FMRP isoforms [15, 16]. At the protein level, we explored for the first time FMRP expression in thymus extracts. We detected different FMRP isoforms (Fig. 2D). The most well-known, the full-length isoform, and two spliced isoforms (bands 1–3 (isoforms 6, 4 and 7) between 71 and 68 KDa) [17], and also three isoforms with a lower molecular weight (bands 4–6). Comparing male and female healthy donors, we observed a higher expression in males for the three isoforms with a high molecular weight (Fig. 2D, E). This could reflect the higher expression of *FMR1* mRNA observed in males (Fig. 2A). The three other isoforms were expressed at the same level (Fig. 2D, E). We then analyzed the level of mRNA expression of transcription factors known to regulate *FMR1* expression, such as Nrf-1 (nuclear respiratory factor 1), Sp1 (specificity protein 1), USF1 (upstream transcription factor 1), MAX (Myc-associated factor X), CTCF (CCCTC-binding factor) [18, 19]. In normal thymuses, only *CTCF* mRNA expression was differently expressed in males and females, and significantly correlated with *FMR1* mRNA expression (Fig. 2B, C).

Our team previously showed a significantly decreased expression of *FMR1* mRNA in the thymus of MG patients whatever the degree of follicular hyperplasia [11]. This decrease was confirmed on a larger number of individuals (fold-change decrease of 1.9 (*p* = 0.0102 for 12 controls and 12 MG patients)). At the protein level, comparing healthy and MG females, we surprisingly observed a decreased expression of the three isoforms with lower molecular weights but not of the isoforms with a high molecular weight. This suggests that the decreased expression of FMR1 mRNA observed in MG [11] could affect the expression of the FMRP isoforms with a low molecular weight (Fig. 2D, E). In MG thymuses, *CTCF* and *MAX* mRNA expression were decreased and their expression was strongly correlated with *FMR1* expression (Fig. 2F, G). *Nrf-1* and *Sp1* mRNA expression were not altered in MG thymuses (data not shown). *USF1* mRNA expression was increased in MG thymuses but not correlated to *FMR1* mRNA expression (data not shown). We previously demonstrated that *FMR1* decreased expression in MG thymuses was observed in thymic epithelial cells (TECs) [11]. We thus investigated the effects of inflammatory molecules on *FMR1* expression in primary TEC cultures. We demonstrated that Poly(I:C) decreased *FMR1* mRNA while IFN-I and IL-6 increased it, and IFN-II had no effect (Fig. 2H). As in MG thymuses, the decreased expression of *FMR1* mRNA in TECs is associated with a decreased expression of *CTCF* and *MAX* mRNA induced by Poly(I:C). In contrast, the increase in *FMR1* expression by IFN-I and IL-6 seemed more or less associated with *CTCF* and *MAX* mRNA increased expression (Fig. 2I, J).

## Discussion

As *FMR1* expression is decreased in MG thymuses, we hypothesized that MG patients may have an abnormal number of CGG triplets, potentially being in the “gray zone”. Analyzing thymic DNA, taking into account the sex, the different alleles of the X chromosome, and the interruption of CGGs by AGG sequences, we did not observe any differences between MG patients and control donors that could been related to a predisposition to MG.

So, how can we explain the decrease of *FMR1* mRNA expression in the thymus of MG patients? While *FMR1* expression has been studied in FXS and FX-related syndromes, little is known about the regulation of its expression in normal condition. Different transcription factors have been implicated in the regulation of *FMR1* expression such as Nrf-1, Sp1, USF1/2, MAX, CTCF [18, 19]. In the thymus, we observed that *FMR1* expression could be related to a predisposition to autoimmunity at two levels. First, in normal thymuses, CTCF controlled its expression in a sex-dependent manner. This observation is very important knowing that females are more prone to develop autoimmune diseases [20]. Second, in the inflammatory environment of MG thymus, *CTCF* and *MAX* were differentially expressed and could be involved in the decreased expression of *FMR1*. The implication of MAX in the regulation of *FMR1* expression in MG thymuses was not clear, as MAX has been shown to repress Nrf1 and Sp1 activation of *FMR1* [18]. *CTCF* is a widely expressed transcriptional regulator implicated in many important processes. In the thymus, CTCF could thus play a central role in regulating *FMR1* expression in normal and inflammatory conditions [21, 22]. In addition, at the protein level, the analysis of FMRP expression is also complicated. The unique FMR1 transcript undergoes alternative splicing, resulting in potentially 20 different FMRP isoforms that have not all been yet characterized [15, 16]. The differences in *FMR1* mRNA expression observed between males and females altered the expression of the most-well known isoforms with high molecular weight. In contrast, the decreased expression of *FMR1* mRNA in MG patients seemed to modify the expression of low molecular weight isoforms. As the expression of thymic FMRP isoforms has never been studied before, our results open up many questions that would need to be investigated further.

Here, we demonstrated that *FMR1* expression in TECs was sensitive to inflammatory signals. Indeed, Poly(I:C) decreased *FMR1*, while IFN-I and IL-6 increased it. These modulations of *FMR1* expression were also associated with *CTCF* and *MAX* expression. Poly(I:C) is known to induce thymic changes, mediated by IFN-β (an IFN-I subtype), that can even lead to MG in mouse [23]. Poly(I:C) induces IFN-I and IL-6 expression in TECs [23]. Here, the opposite effects of Poly(I:C) and IFN-I were surprising, but suggest that Poly(I:C) could induce *FMR1* decrease by a more potent independent signaling pathway or by interfering with the IFN-I and IL-6 signalization. The fact that *FMR1* is well expressed in the thymus [7] and that its expression is influenced by environmental factors strongly suggest that it could play a critical role in the thymus and the immune system. However, the role of FMR1 in relation to immune functions has hardly been explored. Altered number and/or function of regulatory T cells (CD4^+^CD25^+^ T cells) are known to be associated with autoimmune diseases, including MG [24]. Careaga et al*.* showed that human premutation carriers have a decreased level of T cells expressing CD25^+^ upon stimulation in vitro [25]. PBMCs from human premutation carriers display a decreased expression of pro-inflammatory cytokines [25] and an increased expression of the anti-inflammatory cytokine, IL-10 [26]. However, the level of *FMR1* expression has not been analyzed in these studies. In PBMCs from FXS patients, for which FMR1 expression is inhibited, an altered immune response is observed in response to the activation of the LPS and glutamate receptor pathways [27]. In a Drosophila model *Fmr1* mutants exhibit reduced bacterial engulfment, an early step in phagocytosis, and delayed processes that require phagocytosis by glial cells. These data suggest that Fmr1 could regulate the activation of phagocytic immune cells [28]. In addition, gene expression analyses in the brain of *fmr1* knockout mice reveal an over-representation of immunological signaling pathways [29]. The investigation of gene expression in this mouse model could be of interest to decipher the role of FMR1 in the human thymus.

There is clearly a link between *FMR1* and autoimmunity depending on the number of upstream CGG triplets as observed in premutated carriers and FXPOI [8, 9]. Here, we suggest for the first time another link between *FMR1* and autoimmunity at the thymus level. In particular, we showed that FMR1 expression is highly susceptible to the inflammatory environment. Nevertheless, further investigations are necessary to better understand its role in the immune system.

## Data Availability

The datasets used and/or analyzed during the current study are available from the corresponding author.

## Abbreviations

CTCF: CCCTC-binding factor
FMR1: Fragile X mental retardation 1
FMRP: Fragile mental retardation protein
FXS: Fragile X syndrome
MAX: Myc-associated factor X
MG: Myasthenia gravis
PBMC: Peripheral mononuclear blood cell
TEC: Thymic epithelial cell

## References

[1] Fu YH, Kuhl DPA, Pizzuti A, Pieretti M, Sutcliffe JS, Richards S (1991). Variation of the CGG repeat at the fragile X site results in genetic instability: Resolution of the Sherman paradox. Cell.

[2] Hall DA (2014). In the Gray Zone in the Fragile X Gene: What are the Key Unanswered Clinical and Biological Questions?. Tremor Other Hyperkinet Mov (N Y).

[3] Gleicher N, Weghofer A, Barad DH (2012). Cutting edge assessment of the impact of autoimmunity on female reproductive success. J Autoimmun.

[4] Mailick MR, Hong J, Rathouz P, Baker MW, Greenberg JS, Smith L (2014). Low-normal FMR1 CGG repeat length: Phenotypic associations. Front Genet.

[5] Wang Q, Barad DH, Darmon SK, Kushnir VA, Wu YG, Lazzaroni-Tealdi E (2018). Reduced RNA expression of the FMR1 gene in women with low CGG n < 26 repeats. PLoS ONE.

[6] Malecki C, Hambly BD, Jeremy RW, Robertson EN (2020). The RNA-binding fragile-X mental retardation protein and its role beyond the brain. Biophys Rev Biophysical Reviews.

[7] Hinds HL, Ashley CT, Sutcliffe JS, Nelson DL, Warren ST, Housman DE (1993). Tissue specific expression of FMR–1 provides evidence for a functional role in fragile X syndrome. Nat Genet.

[8] Persani L, Rossetti R, Cacciatore C, Bonomi M (2009). Primary Ovarian Insufficiency: X chromosome defects and autoimmunity. J Autoimmun.

[9] Winarni TI, Chonchaiya W, Sumekar TA, Ashwood P, Morales GM, Tassone F (2012). Immune-mediated disorders among women carriers of fragile X premutation alleles. Am J Med Genet A.

[10] Berrih-Aknin S, Le Panse R (2014). Myasthenia gravis: A comprehensive review of immune dysregulation and etiological mechanisms. J Autoimmun.

[11] Cron MA, Maillard S, Deslile F, Samson N, Truffault F, Foti M (2018). Analysis of microRNA expression in the thymus of Myasthenia Gravis patients opens new research avenues. Autoimmun Rev.

[12] Kyewski B, Klein L (2006). A central role for central tolerance. Annu Rev Immunol.

[13] Pretto DI, Mendoza-Morales G, Lo J, Cao R, Hadd A, Latham GJ (2014). CGG allele size somatic mosaicism and methylation in FMR1 premutation alleles. J Med Genet.

[14] Nolin SL, Sah S, Glicksman A, Sherman SL, Allen E, Berry-Kravis E (2013). Fragile X AGG analysis provides new risk predictions for 45–69 repeat alleles. Am J Med Genet Part A.

[15] Brackett DM, Qing F, Amieux PS, Sellers DL, Horner PJ, Morris DR. Fmr1 Transcript Isoforms: Association with Polyribosomes; Regional and Developmental Expression in Mouse Brain. PLoS One. 2013; 8: e58296. 10.1371/journal.pone.0058296 PMC3591412 23505481

[16] Yang WJ, Yang WJ, Yan AZ, Xu YJ, Guo XY, Guo XY (2020). Further identification of a 140bp sequence from amid intron 9 of human FMR1 gene as a new exon. BMC Genet.

[17] Zhang J, Wang G, He W-W, Losh M, Berry-Kravis E, Funk WE (2019). Expression and Characterization of Human Fragile X Mental Retardation Protein Isoforms and Interacting Proteins in Human Cells. Proteomics Insights.

[18] Kumari D, Usdin K. Interaction of the transcription factors USF1, USF2, and α-Pal/Nrf-1 with the FMR1 promoter. Implications for Fragile X mental retardation syndrome. J Biol Chem. 2001;276:4357–64. 10.1074/jbc.M009629200 11058604

[19] Lanni S, Goracci M, Borrelli L, Mancano G, Chiurazzi P, Moscato U, et al. Role of CTCF protein in regulating FMR1 locus transcription. PLoS Genet. 2013;9:e1003601. 10.1371/journal.pgen.1003601 PMC3715420 23874213

[20] Markle JG, Fish EN (2014). SeXX matters in immunity. Trends Immunol.

[21] Herzig Y, Nevo S, Bornstein C, Brezis MR, Ben-Hur S, Shkedy A (2017). Transcriptional programs that control expression of the autoimmune regulator gene Aire. Nat Immunol.

[22] Stik G, Vidal E, Barrero M, Cuartero S, Vila-casadesús M, Mendieta-esteban J, et al. CTCF is dispensable for immune cell transdifferentiation but facilitates an acute inflammatory response. Nat Genet [Internet]. Springer US; 2020;52: 655–661. 10.1038/s41588-020-0643-0 32514124

[23] Cufi P, Dragin N, Weiss JM, Martinez-Martinez P, De Baets MH, Roussin R (2013). Implication of double-stranded RNA signaling in the etiology of autoimmune myasthenia gravis. Ann Neurol.

[24] Balandina A, Lecart S, Dartevelle P, Saoudi A, Berrih-Aknin S (2005). Functional defect of regulatory CD4(+)CD25+ T cells in the thymus of patients with autoimmune myasthenia gravis. Blood.

[25] Careaga M, Rose D, Tassone F, Berman RF, Hagerman R, Ashwood P (2014). Immune dysregulation as a cause of autoinflammation in fragile X premutation carriers: Link between FMRI CGG repeat number and decreased cytokine responses. PLoS ONE.

[26] Marek D, Papin S, Ellefsen K, Niederhauser J, Isidor N, Ransijn A (2012). Carriers of the fragile X mental retardation 1 (FMR1) premutation allele present with increased levels of cytokine IL-10. J Neuroinflammation.

[27] Careaga M, Noyon T, Basuta K, Van de Water J, Tassone F, Hagerman RJ, et al. Group I metabotropic glutamate receptor mediated dynamic immune dysfunction in children with fragile X syndrome. J Neuroinflammation. 2014;11:1–10. 10.1186/1742-2094-11-110 PMC4107617 24942544

[28] O’Connor RM, Stone EF, Wayne CR, Marcinkevicius EV, Ulgherait M, Delventhal R (2017). A Drosophila model of Fragile X syndrome exhibits defects in phagocytosis by innate immune cells. J Cell Biol.

[29] Prilutsky D, Kho AT, Palmer NP, Bhakar AL, Smedemark-Margulies N, Kong SW (2015). Gene expression analysis in Fmr1KO mice identifies an immunological signature in brain tissue and mGluR5-related signaling in primary neuronal cultures. Mol Autism.

